# Association between Health Eating Index-2015 and prostate-specific antigen levels: a cross-sectional study of NHANES 2001–2004

**DOI:** 10.29219/fnr.v69.12639

**Published:** 2025-09-25

**Authors:** Xingpeng Di, Jie Zhang, Liyuan Xiang, Xin Wei, Banghua Liao

**Affiliations:** 1 Department of Urology and Institute of Urology (Laboratory of Reconstructive Urology), West China Hospital, Sichuan University, Chengdu, People’s Republic of China; 2 Department of Clinical Research Management, West China Hospital, Sichuan University, Chengdu, People’s Republic of China

**Keywords:** prostate cancer, prostate-specific antigen, Health Eating Index, National Health and Nutrition Examination Survey

## Abstract

**Objectives:**

Prostate cancer is the most common carcinoma among men worldwide. To elaborate the effect of dietary quality on prostate-specific antigen (PSA), we investigated the association between Health Eating Index-2015 (HEI-2015) and PSA concentration from National Health and Nutrition Examination Survey.

**Methods:**

This cross-sectional analysis of men aged 40 years and older was enrolled from the year 2001–2004. Weighted multivariable logistic and linear regression models were employed to evaluate the association between the HEI-2015 and PSA level.

**Results:**

A total of 1,467 males were enrolled in the study. The results demonstrated that a higher HEI-2015 score was associated with a lower PSA level in the fully-adjusted model (β = –0.388, 95% Confidence interval (CI) = –0.746 to –0.030, *P* = 0.030). Specifically, the consumption of seafood and plant proteins group was found to have an inverse correlation with PSA levels (β = –0.049, 95% CI = –0.088 to –0.009, *P* = 0.020).

**Conclusion:**

Our findings indicate that a higher HEI-2015 score is associated with a reduced risk of PSA among adult men aged 40–55 years in the United States. Furthermore, race, body mass index (BMI), and alcohol drinking may be modifiers of the relationship.

## Popular scientific summary

- Research on the association between HEI-2015 and PSA levels is absent.
- A higher HEI-2015 score is associated with reduced PSA levels among adult men aged 40–55 years.
- Race, body mass index (BMI), and alcohol drinking may be modifiers of the relationship.

Prostate cancer (PCa) is the most prevalent form of cancer and ranks fifth among the leading causes of cancer-related deaths in men worldwide (1). Despite the identification of multiple therapeutic targets, PCa remains a complex urological issue with significant morbidity and mortality (2). Studies demonstrated that lifestyle, specific genetic heritage, physical activity, and dietary food intake were related to PCa (3, 4).

Prostate-specific antigen (PSA) is the most common used biomarker for PCa and enables the assessment of tumor-associated risk (5). Additionally, PSA serves as a trend marker for monitoring the progression of PCa. With advancements in PCa detection methods and increased awareness among the general population, localized PCa at early stages is being diagnosed more frequently. According to the guidelines from the American Urological Association (AUA), active surveillance of PSA levels is a management strategy for watchful waiting, which can delay surgery for people at minimal risk of progression or with a short life expectancy (6). Despite its high sensitivity and accuracy in detecting PCa, PSA levels can be affected by various factors, such as transurethral operation, digital rectal examination (DRE), prostatitis, and biopsy of the prostate before the detection of PSA. Moreover, studies revealed that benign prostate hyperplasia (BPH), demographic characteristics (e.g. age, ethics), energy intake, and antibiotics application could affect PSA levels as well (7).

Intriguingly, the incidence of PCa varies across different regions. Numerous studies have shown that dietary intake (e.g. fat, protein, carbohydrates, vegetables, fish) can influence the development and progression of PCa (8, 9). However, clinical trials have reported conflicting results and few studies have investigated the association between the overall diet quality and the PCa incidence. Recently, the quality of diet has been identified as a factor that affects PCa progression. Studies revealed that high-quality diet intake might reduce the risk of Gleason-grade progression of PCa (10). The Health Eating Index-2015 (HEI-2015), an updated version with improved validity and consistency, was developed by the 2015–2020 Diary Guidelines for Americans (DGA) to assess the effects of food quality on US adults (11). In men with a PSA testing history, high HEI-2005 and alternative HEI-2010 indicated a low risk of PCa. Furthermore, the fish component of the alternative Mediterranean diet score was inversely associated with PCa risk (12).

Given the complex and undetermined mechanisms linking dietary habits to the incidence of PCa, there is a need for further studies to better understand the role of dietary patterns in PCa. In this study, we performed an analysis based on the National Health and Nutrition Examination Survey (NHANES) of the United States (US), trying to investigate the association between the HEI-2015 score and PSA concentrations in men adults. We aimed to provide a novel insight on the early-stage screening and prevention of PCa.

## Materials and methods

### Study population

The NHANES database is designed to assess the health and nutrition status of the US population every 2 years. The NHANES sample is selected from a larger group across the US to represent all the US population. The information of every participants is from interview questions regarding health in the mobile exam center, blood sample and dental examination in the center. In this study, we recruited a total of 10,301 male participants aged 40 years or above from the period spanning 2001–2004. The exclusion criteria include (13–15): (1) age < 40 years old; (2) recent prostate operation history (i.e. prostate biopsy in 4 weeks, DRE in 1 week, cystoscopy operation in 4 weeks); (3) prostate inflammation (i.e. prostatitis); (4) history of PCa; (5) missing PSA and HEI-2015 data; (6) overall energy extreme data (>8,000 kcal per day and <500 kcal per day); (7) missing covariates data (i.e. demographic data, smoking history, alcohol drinking history, coronary heart disease). Finally, 1,467 male participants with intact data were enrolled. It is worth noting that the research protocol of the NHANES dataset adhered to the Helsinki Declaration of the World Medical Association and did not require registration. Additionally, all research protocols were approved by the research ethics review board of the National Center for Health Statistics, and informed consent was obtained from all participants. This study has been reported in line with the STROCSS criteria (16).

### Estimation of diet quality

The dietary intake data in this study was obtained through two 24-h recall interviews conducted by experienced interviewers. To mitigate potential biases introduced by phone interviews conducted 3–10 days later, we specifically utilized data from the first 24-h face-to-face interview in the mobile center by Centers for Disease Control and Prevention (CDC). This approach was chosen to ensure the reliability and accuracy of the dietary intake information for our study’s outcomes.

The diet quality was measured by HEI-2015 and its components (nine adequacy components and four moderation components), which were scored from 0 to 100. The energy for each food was evaluated and categorized utilizing the Food and Nutrient Database for Dietary Studies/Food Patterns Equivalents Database from the United States Department of Agriculture (FNDDS/FPED). The more adequate component intake indicated healthier diet quality. The less moderation component intake indicated better diet quality. These components were evaluated as amounts per 1,000 kcal except for fatty acids (unsaturated/saturated fats), saturated fats (% energy), and added sugars (% energy).

### Assessment of PSA level

The outcome was the concentration of PSA (ng/mL). A Beckman Access utilizing the Hybritech PSA method was used for addressing the serum samples to record serum total PSA level (https://wwwn.cdc.gov/Nchs/Nhanes/2001-2002/L11P_2_B.htm). And the ratio of free PSA and total PSA was also collected for assessing the risk of PCa.

### Covariates

To better clarify the correlation between HEI-2015 and PSA, several covariates were selected from demographics (e.g. age, education level, and ethnicity), laboratory exams (e.g. diabetes), physical examinations (e.g. body mass index [BMI], kg/m^2^), and questionnaires (e.g. smoking history, alcohol drinking history). In detail, the categorial variable included age (40–55, 56–70, and 71–85 years old), races (Mexican American, non-Hispanic Black and White, other Hispanic and races), education level (less than 12th grade, high school grade, and college grade), marital state (married, divorced, widowed, separated, never married, and living with a partner), smoking history, hypertension (average blood pressure over 140/90 mmHg), and coronary heart disease, diabetes mellitus with or without diabetes medication based on the guidelines were included (17). The continuous variables included family income-to-poverty ratio (≤1, 1–2, >2), BMI (20 kg/m^2^, 20–25 kg/m^2^, over 30 kg/m^2^), and alcohol drinking history (≤1 drink per week, 1–2 drinks per week, and three or more drinks per week).

### Statistical analysis

The statistical analysis was conducted using recommended sampling weights, strata, and primary sample units as outlined by the CDC. To account for the skewness of the serum PSA concentration data, a log2 transformation was applied before conducting further analysis (as previously described in reference (13)). Continuous variables were reported as mean ± standard deviation (SD), while categorical variables were presented as proportions or counts. The chi-square model was used to calculate the proportions. The HEI-2015 score was categorized into four quartiles, denoted as Q1–Q4 (Q1: 20.8–42.9, Q2: 43.0–51.0, Q3: 51.0–59.8, Q4: 59.8–90.6), for subsequent analysis. Weighted linear and multivariate logistic regression models were employed to investigate the relationship between HEI-2015 and PSA concentration, with and without adjustment for covariates.

Weighted linear or multivariate logistic regression models were employed to investigate the relationship between HEI-2015 and PSA concentration, with and without adjustment for covariates. The crude model did not include any adjustments. Model 1 was adjusted for demographic characteristics, including age, race, education level, family income-to-poverty ratio and marital status. Model 2 further adjusted for additional covariates such as total energy intake, BMI, smoking history, alcohol drinking history, diabetes mellitus, hypertension, and coronary heart disease.

Moreover, the association between HEI components and age-stratified PSA levels was analyzed using a multinomial logistic regression model. Subgroup and interactive analyses were conducted to identify variables that potentially modified the correlation between HEI-2015 and PSA. To explore possible non-linear associations in age-stratified groups, spline smoothing with a generalized additive model (GAM) was performed.

The *nhanesR* package of *R* software version 4.1 (http://www.R-project.org; The R Foundation) and EmpowerStats (http://www.empowerstats.com, X&Y Solutions, Inc.) were used. *P* < 0.05 (two-tailed) indicated statistically significant.

## Results

### Baseline characteristics of participants

**Fig. 1:**
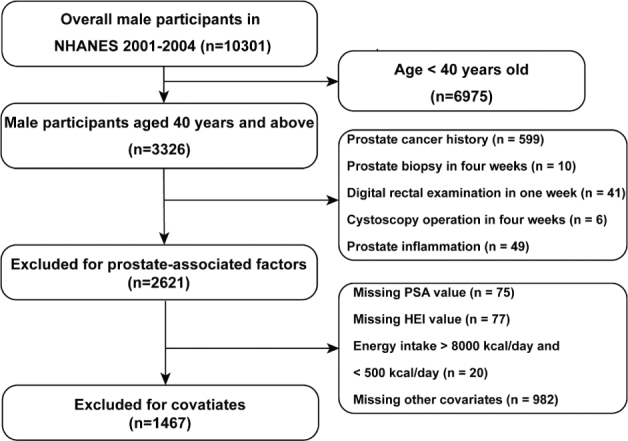
Flow diagram of participants screening. HEI-2015: Health Eating Index-2015; NHANES: National Health and Nutrition Examination Survey; PSA: prostate-specific antigen.

A total of 1,467 male participants aged 40 years old and above were enrolled (Fig. 1). The baseline information divided by Q1–Q4 of the HEI-2015 score were shown in Table 1. The higher dietary quality quartile of the participants tended to be older, had higher education levels, lower diabetes, and had more than three drinks of alcoholic beverage, and higher PSA concentration (log2 transformed). The weighted distribution of race, education level, family income-to-poverty ratio, alcohol drinking, smoking, diabetes, hypertension, and coronary heart disease was similar (*P* > 0.05).

**Table 1:** Characteristics of participants by categories of Healthy Eating Index 2015: NHANES 2001–2004, weighted

Characteristics	HEI-2015				*P*
	Q1 (20.8–42.9)	Q2 (43.0–51.0)	Q3 (51.0–59.8)	Q4 (59.8–90.6)	
Number (*n*)	367	366	367	367	
tPSA (Log_2_ transformation, ng/mL)	−0.1 ± 1.3	0.1 ± 1.4	0.1 ± 1.3	0.2 ± 1.3	0.766
f/t PSA (%)	30.3 ± 13.7	29.6 ± 12.1	29.5 ± 11.7	30.8 ± 13.0	0.587
HEI-2015 (continuous)	36.4 ± 4.8	47.2 ± 2.3	55.1 ± 2.5	67.9 ± 6.3	<0.01**
Age (years)	55.0 ± 11.7	56.8 ± 12.6	57.4 ± 13.2	60.4 ± 12.6	<0.01**
Race (*n*/%)					0.891
Mexican American	73 (19.9%)	71 (19.4%)	73 (19.9%)	78 (21.3%)	
Non-Hispanic Black	72 (19.6%)	63 (17.2%)	52 (14.2%)	52 (14.2%)	
Non-Hispanic White	209 (56.9%)	215 (58.7%)	222 (60.5%)	216 (58.9%)	
Other Hispanic and races	13 (3.5%)	17 (4.6%)	20 (5.4%)	21 (5.7%)	
Education level (*n*/%)					<0.01**
Lower than 12th grade	110 (30.0%)	95 (26.0%)	92 (25.1%)	80 (21.8%)	
High school grade	99 (27.0%)	94 (25.7%)	75 (20.4%)	71 (19.3%)	
College grade	158 (43.1%)	177 (48.4%)	200 (54.5%)	216 (58.9%)	
Family income-to-poverty ratio (*n*/%)					0.063
≤ 1	59 (16.1%)	49 (13.4%)	38 (10.4%)	34 (9.3%)	
> 1, ≤ 2	80 (21.8%)	71 (19.4%)	74 (20.2%)	62 (16.9%)	
> 2	228 (62.1%)	246 (67.2%)	255 (69.5%)	271 (73.8%)	
Marital state (*n*/%)					0.280
Married	250 (68.1%)	266 (72.7%)	246 (67.0%)	272 (74.1%)	
Divorced	36 (9.8%)	35 (9.6%)	40 (10.9%)	35 (9.5%)	
Widowed	19 (5.2%)	23 (6.3%)	27 (7.4%)	20 (5.4%)	
Separated	10 (2.7%)	7 (1.9%)	8 (2.2%)	7 (1.9%)	
Living with partner	28 (7.6%)	13 (3.6%)	20 (5.4%)	13 (3.5%)	
Never married	24 (6.5%)	22 (6.0%)	26 (7.1%)	20 (5.4%)	
BMI (kg/m^2^, *n*/%)					0.065
≤ 20	9 (2.5%)	8 (2.2%)	8 (2.2%)	7 (1.9%)	
> 20, ≤ 25	63 (17.2%)	83 (22.7%)	83 (22.6%)	94 (25.6%)	
> 25, ≤ 30	176 (48.0%)	160 (43.7%)	171 (46.6%)	179 (48.8%)	
> 30	119 (32.4%)	115 (31.4%)	105 (28.6%)	87 (23.7%)	
Alcohol drinking history (drinks/week)					<0.01**
≤ 1	225 (61.3%)	200 (54.6%)	206 (56.1%)	183 (49.9%)	
1–2	50 (13.6%)	44 (12.0%)	34 (9.3%)	39 (10.6%)	
≥ 3	92 (25.1%)	122 (33.3%)	127 (34.6%)	145 (39.5%)	
Smoking history (*n*/%)					0.248
Non-smoker	104 (28.3%)	113 (30.9%)	129 (35.1%)	134 (36.5%)	
Smoker	263 (71.7%)	253 (69.1%)	238 (64.9%)	233 (63.5%)	
Diabetes mellitus (*n*/%)					0.042*
No	309 (84.2%)	320 (87.4%)	300 (81.7%)	302 (82.3%)	
Yes	58 (15.8%)	46 (12.6%)	67 (18.3%)	65 (17.7%)	
Hypertension (*n*/%)					0.548
No	194 (52.9%)	170 (46.4%)	187 (51.0%)	169 (46.0%)	
Yes	173 (47.1%)	196 (53.6%)	180 (49.0%)	198 (54.0%)	
Coronary heart disease (*n*/%)					0.795
No	340 (92.6%)	336 (91.8%)	333 (90.7%)	334 (91.0%)	
Yes	27 (7.4%)	30 (8.2%)	34 (9.3%)	33 (9.0%)	

Mean ± SD for continuous variables, *P*-value was by survey-weighted linear regression. % for categorical variables, *P*-value was by survey-weighted Chi-square test. Q1 represents the unhealthiest diet quality, Q4 represents the healthiest diet quality. HEI-2015: Health Eating Index-2015; tPSA: total prostate-specific antigen; f/t PSA: free/total prostate-specific antigen; BMI: body mass index; NHANES: National Health and Nutrition Examination Survey; SD: standard deviation.

* *P* < 0.05,

** *P* < 0.01.

### The association between HEI-2015 score and PSA level

**Fig. 2:**
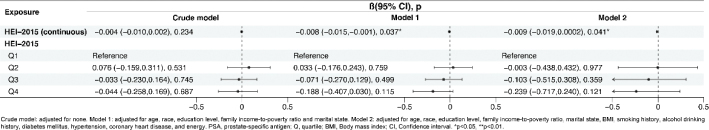
Association between Healthy Eating Index 2015 (HEI-2015) and PSA level, weighted. Crude model: adjusted for none. Model 1: adjusted for age, race, education level, family income-to-poverty ratio and marital state. Model 2: adjusted for age, race, education level, family income-to-poverty ratio, marital state, BMI, smoking history, alcohol drinking history, diabetes mellitus, hypertension, coronary heart disease, and energy. PSA: prostate-specific antigen; Q: quartile; BMI: body mass index; CI: confidence interval. *P < 0.05, **P < 0.01.

Figure 2 shows the association between HEI-2015 score and PSA concentration (log2 transformed). Significant associations were found between continuous HEI-2015 score and PSA level in model 1 (β = –0.008, 95% Confidence interval [CI] = -0.015 to –0.001, *P* = 0.037) and model 2 (β = –0.009, 95% CI = -0.019 to 0.0002, *P* = 0.041). However, no such associations were found between different quartiles of HEI-2015 and PSA level compared with Q1.

**Fig. 3:**
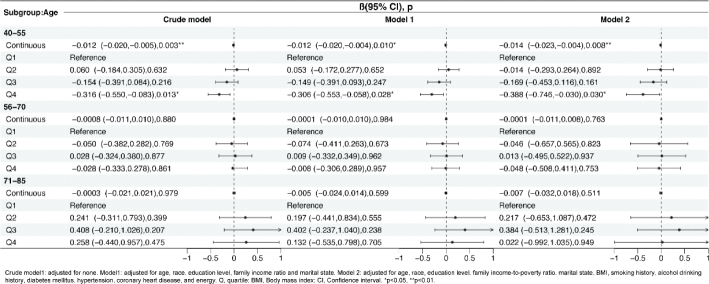
Age stratified analyses by the age-stratified linear regression model, weighted. Crude model1: adjusted for none. Model1: adjusted for age, race, education level, family income ratio and marital state. Model 2: adjusted for age, race, education level, family income-to-poverty ratio, marital state, BMI, smoking history, alcohol drinking history, diabetes mellitus, hypertension, coronary heart disease, and energy. Q: quartile; BMI: body mass index; CI: confidence interval. *P < 0.05, **P < 0.01.

Furthermore, age stratified logistic regression analyses demonstrated a relationship between continuous HEI-2015 score and PSA level in men aged 40–55 years old in model 2 (β = –0.014, 95% CI = -0.023 to –0.004, *P* = 0.008) (Fig. 3). Compared with Q1 of HEI-2015 score, Q4 level of HEI-score was related to lower PSA level in men aged 40–55 (Model 2, β = –0.388, 95% CI = -0.746 to -0.030, *P* = 0.030).

### HEI-2015 components and PSA level in 40–55 years old population

**Fig. 4:**
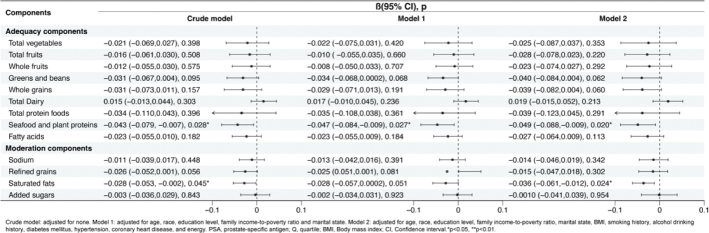
Association of Healthy Eating Index 2015 (HEI-2015) components with PSA concentration (Log2 transformed) aged 40–55 years old, weighted. Crude model: adjusted for none. Model 1: adjusted for age, race, education level, family income-to-poverty ratio and marital state. Model 2: adjusted for age, race, education level, family income-to-poverty ratio, marital state, BMI, smoking history, alcohol drinking history, diabetes mellitus, hypertension, coronary heart disease, and energy. PSA: prostate-specific antigen; Q: quartile; BMI: body mass index; CI: confidence interval. *P < 0.05, **P < 0.01.

We further examined the association between HEI-2015 components and PSA concentration (log2 transformed) in the 40–55 years old group. The results demonstrated that the seafood and plant proteins group was inversely correlated with PSA level in the model 1 (β = –0.047, 95% CI = -0.084 to -0.009, *P* = 0.027) and model 2 (β = –0.049, 95% CI = -0.088 to –0.009, *P* = 0.020). No significant difference was found between other HEI-2015 components and PSA level (Fig. 4). Although saturated fats were negatively associated with PSA levels in the crude model and model 2 (*P* < 0.05), there was no such effect in model 1 (*P* > 0.05).

### Subgroups and interactions analyses and spline smoothing

Furthermore, stratified logistic regression indicated that race, BMI, and alcohol drinking history could modulate the relationship between HEI-2015 and PSA level. The interactive *P*-values for race, BMI and alcohol drinking history were <0.01, <0.01, 0.013 (Supplementary Table 1).

Given that associations were found between HEI-2015 and PSA level in 40–55 years old subgroup, smooth curve fitting analysis was further conducted to investigate whether there was the non-linear relationship between HEI-2015 and PSA level in other age subgroups (log2 transformed) (Supplementary Fig. 1). The PSA level increased when the HEI-2015 score was lower than 60. The PSA level was lower when the HEI-2015 score increased to more than 60. The association in the 55–70 years old subgroup seemed stable before the HEI-2015 score of 70 and met a short increase after the 70.

## Discussion

We explored the association between HEI-2015 components and PSA in this cross-sectional study. Our findings revealed that adherence to a healthy diet, as indicated by an HEI-2015 score above 60, was associated with a lower PSA level in men aged 40–55 years. Specifically, the consumption of seafood and plant proteins were negatively associated with PSA levels.

Currently, several studies have investigated the relationship between dietary intake and the risk of PCa. The HEI-2015 has been shown in previous analyses to effectively capture variations in diet quality and reflect the multidimensional nature of healthy eating, with higher levels of validity, reliability, and criterion validity. Consistent with previous studies, a comprehensive prospective analysis revealed a protective effect of a healthier diet in lowering the risk of pancreatic ductal adenocarcinoma (PDAC) (18). Besides the US population, a multicentral and case-control study revealed that the HEI-2015 score was inversely related to the risk of oral and pharyngeal cancer in the Italian population (19). Similar results were also found in squamous cell carcinoma of the lung (20). In addition, a higher HEI-2015 score has been reported to be associated with more anti-inflammatory food intake, which might prevent cancer recurrence (21). Importantly, post-diagnostic adherence to a high-quality diet was associated with cancer mortality of cancer survivors (22).

The HEI-2015 score takes into account both adequacy and moderation components of dietary quality. A higher score reflects a healthier dietary pattern characterized by increased consumption of adequacy components. In this study, we observed that higher intake of seafood and plant proteins was associated with lower PSA concentrations (log2 transformed). Dietary recommendations often suggest a varied diet that includes a high intake of plant foods and protein sources, such as fish (23). Fish, in particular, is rich in omega-3 polyunsaturated fatty acids, which have anti-inflammatory properties and may contribute to a lower prevalence of PCa (24). Epidemiological studies have shown that many Asian countries, where seafood consumption is high, exhibit lower incidence rates of various cancers (25). Furthermore, a meta-analysis of 20 clinical trials provided evidence supporting the protective role of fish consumption in reducing the risk of lung cancer (26). However, it is worth noting that a consortium of 15 cohort studies did not find sufficient evidence to support the protective effect of seafood consumption on PCa (27). Therefore, further in-depth research is needed to explore the potential benefits of different types of fish, including both dark and white meat varieties, which contain varying amounts of omega-3 fatty acids.

Plant proteins, found in plant-based foods rich in dietary fiber, vitamins, minerals, and phytochemicals, have been associated with a reduced risk of various diseases, including diabetes, cancer, and metabolic syndrome (28). Consumption of plant proteins can serve as a substitute for proteins from other sources and has been linked to lower mortality rates. For instance, a dose-response meta-analysis demonstrated that that higher plant protein intake was associated with a lower risk of all-cause mortality (29). Additionally, a large prospective study conducted in Japan suggested that replacing animal protein with plant protein could lead to lower mortality rates (30).

Recent nutrition research, including the analysis of dietary intake data from sources such as the NHANES database, has highlighted the potential influence of diet on disease outcomes. Interestingly, studies have also shown that different dietary qualities can impact the composition of gut microbiota (31). A high-quality diet may act as a modulator for various physiological processes in the human body, potentially affecting tumor pathophysiology. Therefore, our study provides novel insights into the prediction and prevention of PCa. Specifically, the components of the HEI-2015, particularly seafood and plant protein intake, may offer suggestions for reducing all-cause mortality associated with PCa.

Interestingly, we found that the trends in the association between HEI-2015 and PSA levels across different age subgroups are different. The differences may be attributed to prostate volume, age-related changes in diet and metabolism, hormonal alterations and other disease status. These factors are all critical variables affecting this difference.

To our knowledge, our study is the first to analyze the large-scale US population and assess the role of the Healthy Eating Index-2015 (HEI-2015) in PCa. However, there are also some limitations of our study. Firstly, we cannot demonstrate a causal association between HEI-2015 and PSA levels since the NHANES data are cross-sectional designed. Furthermore, the generalizability of our findings to other populations or nations is uncertain. Further research in different populations is needed to validate our results. Secondly, our data collection relied on the first 24-h recall, which may not capture the full extent of dietary intake. Thirdly, potential inaccuracies or missing data in the questionnaire responses may introduce bias and the small sample size caused by data sheltering also affect the confidence of the outcome. Finally, despite our efforts to account for confounding factors in the regression analysis, there may still be variables influencing the outcomes that require further investigation and confirmation.

## Conclusion

In summary, a large-scale cross-sectional analysis from the NHANES dataset demonstrated that a higher HEI-2015 score was associated with lower PSA concentration (log2 transformed) in the US men aged 40–55 years. Race, BMI and alcohol drinking history were potential modifiers of the relationship. Further studies are needed to validate the outcomes.

## Data Availability

All raw data were publicly available at the NHANES database (https://www.cdc.gov/nchs/nhanes/index.htm).
